# Estimation of dust concentration by a novel machine vision system

**DOI:** 10.1038/s41598-022-18036-8

**Published:** 2022-08-11

**Authors:** Hamid Reza Arjomandi, Kamran Kheiralipour, Ali Amarloei

**Affiliations:** 1 Mechanical Engineering of Biosystems Department, Ilam University, Ilam, Iran; 2 Department of Environmental Health, Faculty of Health, Ilam University of Medical Science, Ilam, Iran

**Keywords:** Environmental sciences, Engineering, Optics and photonics

## Abstract

The dust phenomenon is one of the main environmental problems that it reversely affects human health and economical and social activities. In the present research, a novel algorithm has been developed based on image processing to estimate dust concentration. An experimental setup was implemented to create airborne dust with different concentration values from 0 to 2750 µg.m^−3^. The images of the different dust concentration values were acquired and analyzed by image processing technique. Different color and texture features were extracted from various color spaces. The extracted features were used to develop single and multivariable models by regression method. Totally 285 single variable models were obtained and compared to select efficient features among them. The best single variable model had a predictive accuracy of 91%. The features were used for multivariable modeling and the best model was selected with a predictive accuracy of 100% and a mean squared error of 1.44 × 10^−23^. The results showed the high ability of the developed machine vision system for estimating dust concentration with high speed and accuracy.

## Introduction

Dust is small materials that can be observed in the air as suspended particles for some time or as a settled layer on surfaces^1^. The solid particles with a diameter smaller than 100 μm are called dust^2^. The dust sources are soil, sea salt, pollen and spores, fires, volcanic ash, etc. It can be emerged by natural moving or anthropogenic activities such as construction/demolition, minerals extraction, transport, and industrial and agricultural activities^3^.

The dust has undesired effects on various areas such as the environment, industry, agriculture, living animals and plants, and worst of all, human heal^4,5^, so that it causes economic and social damages. Because of these effects, it must be controlled or managed and so the determination of dust concentration must be followed as the first step in this regard^4^.

Conventional devices determine dust concentration in three ways: deposited dust^6–8^, dust soiling^9,10^, and airborne dust^11–17^. Measuring the deposited dust needs gathering the dust from a surface and weighting that so it is a time-consuming method. A reference method for measuring the dust mass concentration is the gravimetric method. This method is simple but it needs a filter^18^. Other dust measuring devices for both dust soiling and airborne dust are expensive methods.

Recently researchers developed different fast systems to measure dust concentration based on laser^19^ and ultrasonic^20^ where these methods are also expensive and cover small points for measurement. Contrariwise, machine vision systems via processing visible images have been found as cheap and useful tools that have area measurements.

Due to low cost and high speed, accuracy, and reliability^21^ the image processing technique has vast applications in different fields through sensing the color, texture, and shape of objects^22–24^. It has different steps including image acquisition, image processing, and data analysis to understand image concepts or measure/control a process^25^. The data extracted from the images (features) are compared to select the efficient information and use them for the prediction goals^22–24^. Machine vision can be used to solve the different control and measurement issues in industry, medicine, agriculture, and natural resources. Researchers showed the ability of image processing technique in measuring and analyzing the grain size and size distribution^26–28^. The results of these researches suggest the development and evaluation of a machine vision system to estimate the dust concentration.

Due to the importance of the determination of dust concentration and the development of simple, fast, and cheap method in this regard, the purpose of the present research was to develop a novel machine vision system to determine the dust concentration in the air. The novelty of the present work is in the point of application of image processing technique to develop a machine vision system as a simple, fast, and cheap technique for estimating dust concentration which has not been reported till now.

## Results

A novel machine vision system based on image processing technique and regression modeling was developed for predicting dust determination in the air.

### Single variable modeling

The different extracted features were used individually to predict the dust concentration by single variable modeling. For this purpose, different single variable models based on linear single-variable regression were developed. Considering all extracted features for all image channels, 285 linear models were obtained. The coefficient of determination (R^2^) of the models was calculated as the predictive accuracy. The models were compared according to the values of their predictive accuracy.

**Figure 1:**
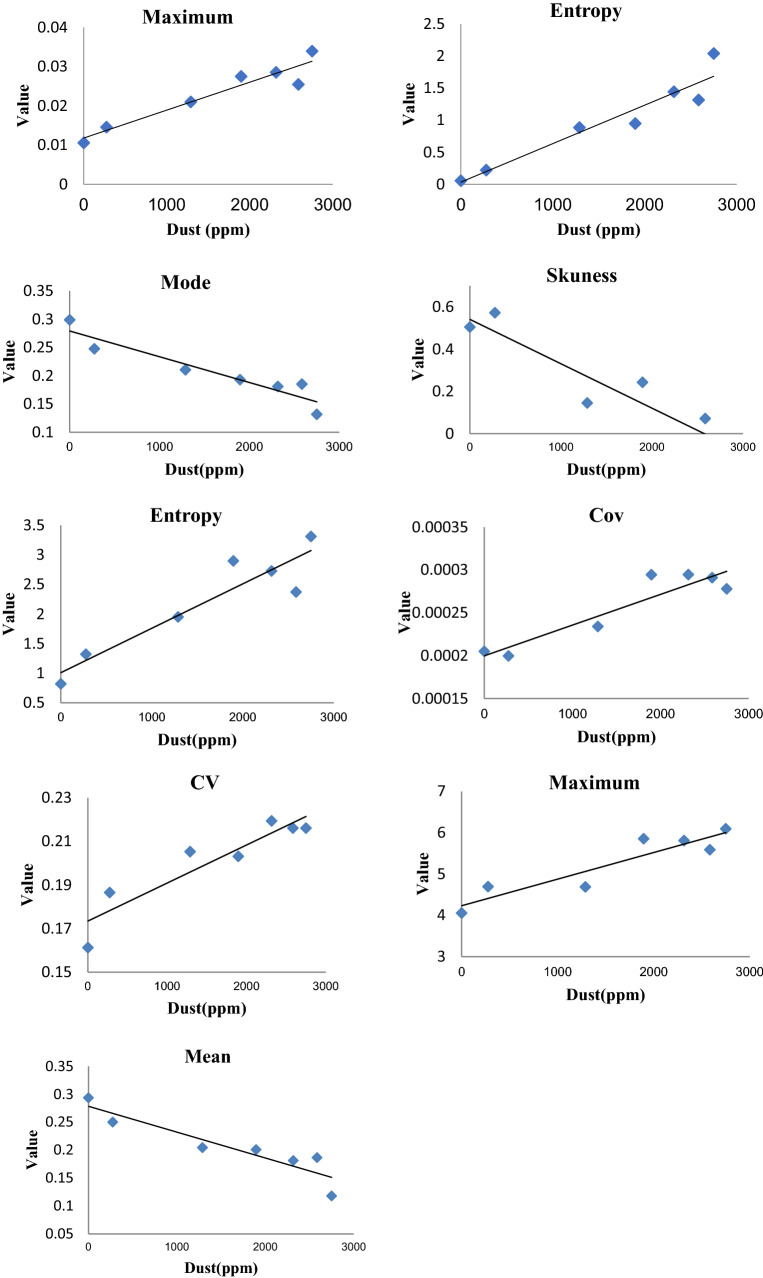
Single variable linear model for predicting dust concentration based on maximum of Cr, entropy of Cr, mod of H, skewness of I3, Entropy of L*, covariance of S, coefficient of variation of H, maximum of b* and mean of H channel.

Among the 285 developed single variable models, those with higher predictive accuracy were selected as better prediction models. Figure 1 and Table 1 shows the selected features and the developed single-variable models. In Fig. 1 and Table 1, y is dust concentration and x is an image feature.

**Table 1:** Single variable models and their predictive accuracy.

No.	Feature	Model*	Accuracy (%)
1	Maximum of Cr channel	$Y=+7E-06X+0.0118$	91.12
2	Entropy of Cr channel	$Y=+6E-04X+0.0352$	91.12
3	Mod of H channel	$Y=-5E-05X+0.2792$	88.55
4	Skewness of I3 channel	$Y=-2E-04X+0.5412$	87.14
5	Entropy of L* channel	$Y=+8E-04X+1.0085$	86.29
6	Covariance of S channel	$Y=+4E-08X+0.0002$	85.88
7	Variation coefficient of H channel	$Y=+2E-05X+0.1734$	85.32
8	Maximum of b* channel	$Y=-6E-04X+4.2342$	85.16
9	Mean of H channel	$Y=-5E-05X+0.2784$	85.03

*Y is the dust concentration and X is feature.

To increase the accuracy of the prediction of dust concentration and thus increase the coefficient of determination of predicting models, different multivariate linear models were developed.

### Multivariable modeling

To have a more accurate and reliable prediction model for dust concentration, multivariate linear models were developed. Table 2 shows eight developed multivariable models and their specifications.

**Table 2:** The specifications of multivariable model to predict dust concentration.

Model no.	No. of variables	a	b	c	d	e	f	g	h	i	j	R^2^ (%)	MSE
1	2	− 69,753.90	774.66	− 686.07	0	0	0	0	0	0	0	92.96	7.35e + 04
2	3	− 71,070.45	846.43	2624.96	1550.57	0	0	0	0	0	0	93.02	7.29e + 04
3	4	− 252,146.54	33.68	− 2470.80	− 1690.73	943.09	0	0	0	0	0	94.22	6.03e + 04
4	5	− 442,462.97	− 1547.58	− 2472.13	− 1064.62	− 3483.86	− 1971.96	0	0	0	0	94.73	5.50e + 04
5	6	583,750.38	2078.25	− 16,097.60	86.41	1944.07	20,293,025.42	3581.75	0	0	0	100	1.7580e − 20
6	7	63,755.41	1059.32	− 23,858.79	− 382.78	520.57	21,346,253.75	− 9437.02	6654.24	0	0	100	3.06e − 22
7	8	76,137.54	1464.15	− 14,129.35	1921.42	− 171.40	24,232,666.28	3514.50	− 642.51	1134.20	0	100	1.44e − 23
8	9	65,288.26	1600.17	− 13,383.43	2166.48	− 117.65	24,312,802.67	5264.11	− 708.58	792.79	458.31	100	3.79e − 22

According to Table 2, the best linear multivariate model was presented in Eq. 1. The value of constant parameters in Eq. 1 were − 71,070.45, 10,464.15, − 14,129.35, 1921.42, − 171.40, 24,232,666.28, 3514.50, − 642.51, and 1134.20 for *a*, *b*, *c*, *d*, *e*, *f*, *g*, *h*, and *i*, respectively.

1 $$DC = a \times MaxCr + b \times AntCr + {\text{c}} \times {\text{ModeH}} + {\text{d}} \times {\text{SkunessI}}3 + {\text{e}} \times {\text{ AntL*}} + {\text{f}} \times {\text{CovS}} + {\text{g}} \times {\text{CvH}} + {\text{h}} \times {\text{Maxb*}} + {\text{i}}$$

where:

DC: the dust concentration,

MaxCr: the maximum of channel Cr,

AntCr: the entropy of channel Cr,

ModeH: the mode of channel H,

SkunessI3: the skewness of channel I3,

AntL*: the entropy of channel L*,

CovS: the covariance of channel S,

CvH: the coefficient of variation of channel H,

Maxb: the maximum of channel b*.

a, b, c, d, e, f, g, h, and i: the constant parameters.

## Discussion

In the present study, images of different dust concentration values were acquired. Using image processing technique and single and multivariable modeling methods, the concentration of the dust was predicted. Among the different extracted color and texture features, some features were selected as optimum features according to the predictive accuracy of the single variable models. According to Fig. 1 and Table 1, the coefficient of determination of the best single variable models was between 85 and 91%. The best models to predict the dust concentration were models based on the maximum and entropy of the Cr channel in comparison with other models due to the higher coefficient of determination (91%).

The selected features were used for multivariable modeling and the best dust concentration predicting model was selected with a coefficient of determination of 100%. The first model in Table 2 includes two variables and one variable was added to each next model based on the forward selection method. Finally, the last model has nine independent variables. The independent variables in these models were those variables in Fig. 1 and Table 1. For example, the independent variables of the first model in Table 2 are the maximum and entropy of the Cr channel. The second model includes the same features as the previous model besides the mod of the H channel. Likewise, the lost model contains all features in Fig. 1 and Table 1.

By comparing the models in Table 2 from Model No. 1 (2-variable) to Model No. 8 (9-variable), the highest coefficient of determination belonged to models No. 5 to 8 which is 100%. But by comparing models No. 5 to 8, the least mean squared error belonged to model No. 7 (8-variable model) which is 1.44 × 10^−23^ (Eq. 1). Therefore, the best model for predicting dust concentration is model No. 7 which has the highest predictive accuracy and the least mean squared error compare to other models in Table 2.

Yu et al.^19^ developed and evaluated a dust measuring system based on the laser. The accuracy of their system was between 0.982 and 0.987 for different dust sizes. Zhang et al.^20^ developed an ultrasonic system to measure dust concentration in the range of 100–900 g/m^3^. They reported that the minimum and maximum errors of the system were 2.56–10.4%, respectively. As the accuracy of the present research is 100% with a low mean squared error of 1.44 × 10^−23^, the results of the present research show the feasibility of the machine vision system to estimate dust concentration with high accuracy.

The results can be used to implement a machine vision system for real-time, fast, and accurate estimation of dust concentration. To this end, the image processing algorithm of the machine vision system can be modified to extract just efficient features (not all features) and putting their values in the optimum model to calculate the dust concentration. The challenge of using this system in the real condition is avoiding the existence of moving objects in front of the camera lens. So, the system must be located in a place without the existence of any moving object for the camera. In future works, smartphones can be used to develop small machine vision systems for measuring dust concentration.

## Methods

The present research has been conducted at the Ilam University, Ilam, Iran. This section includes image acquisition, image processing, and data analysis.

### Image acquisition

In the present study, the experimental setup to capture the images of airborne dust consisted of a glass chamber, a blower, a measuring device, and a camera.

**Figure 2:**
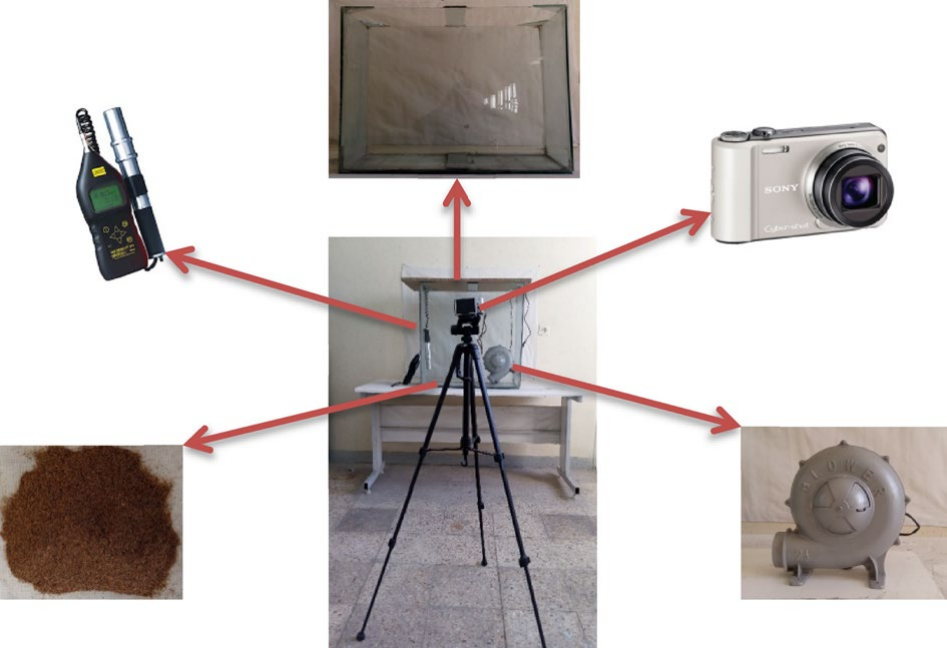
Image acquisition parts.

The chamber was made to create dust storm inside that by a blower. It was made of glass to provide a transparent condition to acquire the dust images from outside of the chamber. The dimensions of the chamber were 70 × 70 × 35 cm^3^ (Fig. 2) and the glass thickness was 5 mm.

A dust meter (model CEL-712 Microdust Pro, Casella Co., UK) with a measurement range of 1–250,000 µm.m^−3^ was used to measure dust concentration in the chamber (clay soil). This dust meter has a calibration container which should be cleaned and calibrated after each use of the probe.

A blower (model GTP03A10, Electric Silver Co., China) with a power of 360 W was used for blowing the clay soil in the glass chamber. Both the electric blower and dust meter were installed inside the chamber (Fig. 2).

The studied dust concentration values in the present research were: 0, 275, 1289, 1896, 2316, 2585 and 2750 µg.m^−3^. To do this, firstly 60 g of clay soil was weighed using an AND Digital Scale (GF-600 Model, Japan) and poured onto the bottom of the glass chamber. To prepare other concentration values, each time 60 g of clay soil was added to the previous amount.

**Figure 3:**
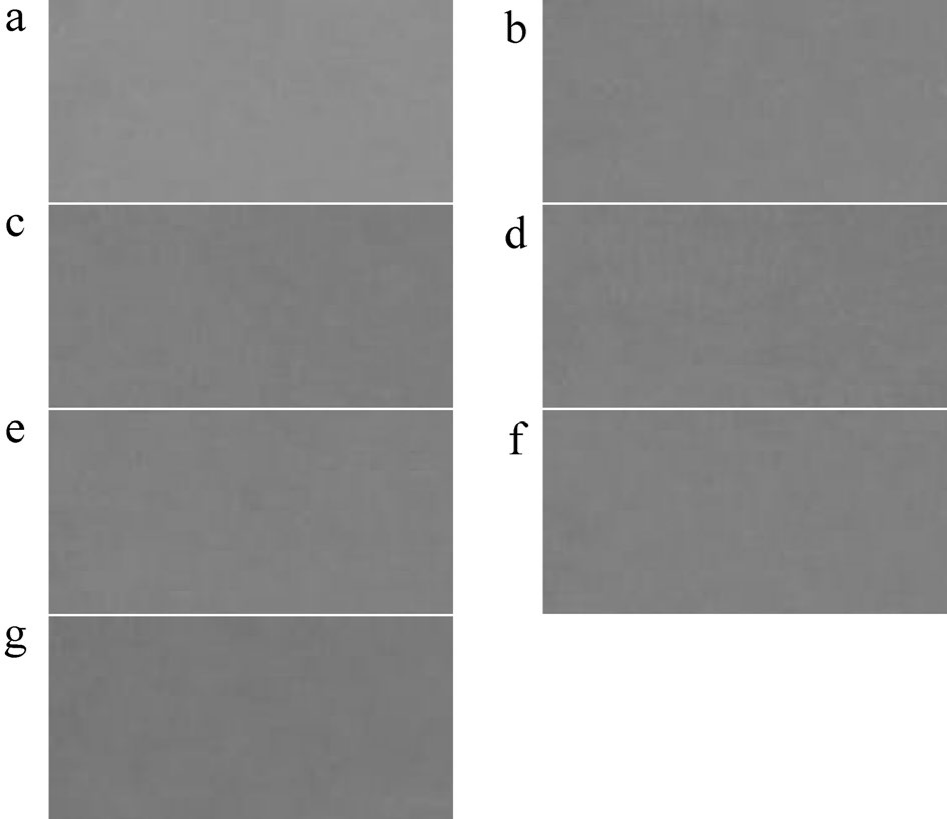
The captured images (left) and the separated segments of the captured images (right) for different dust concentration values: (**a**) 0, (**b**) 275, (**c**) 1289, (**d**) 1896, (**e**) 2316, (**f**) 2585 and (**g**) 2750 µg.m^−3^.

To capture the dust images, a digital camera (Model: DSC-H70, SONY Co., Japan) with 16.1 MPix was used. The camera was placed 1 m far from the glass chamber and the images were acquired 5 times for each dust concentration. The sample images captured by the camera have been presented in Fig. 3.

### Image processing

After the image acquisition step, the images were transferred into a SONY VPC-CMeFGX/B laptop with Intel (R) Core (TM) i5 M520 @ 2.40 GHz processor and 4 GB of soft memory. An image processing algorithm was developed and coded in MATLAB 2013A Software to process the images. In the present study, image processing was performed in two steps including pre-processing and feature extraction.

### Image preprocessing

Image preprocessing is the first step in image analysis and also is the main part of any machine vision system. All operations performed in the preprocessing steps are for the following purposes:

1. Separation of a segment of the image that is appropriate for the image processing step. Figure 2 Right gives useful segments of the images captured for different dust concentration values from 0 to 2750 µg.m^−3^.
2. Convert RGB images to different spaces such as HSV, I1I2I3, L*a*b*, YCrCgCb, NRGB, and gray spaces and separate different channels of each space. This step was conducted based on references^22–24,29^.

Totally 19 image channels including R, G, B, L*, a*, b*, NR, NG, NB, Cr, Cg, Cb, H, S, V, II, I2, I3, and gray levels were obtained by the coded program. These channels were used in the next step to extract some features.

### Feature extraction

An image is made up of many pixels and each of these pixels is presented by a value (pixel value) and a spatial dimension (x, y) in a 3-D matrix. Despite the finite number of pixels in the matrix, it is almost impossible to find the relationship between all the corresponding pixels in several images. For this reason, some important features of the images need to be extracted and analyzed.

As the ability of machine vision is to sense the color and texture of objects^22–24^, the features related to these attributes were extracted. In the present research, different color and texture features were extracted from the obtained image channels including R, G, B, L*, a*, b*, NR, NG, NB, Cr, Cg, Cb, H, S, V, II, I2, I3 and gray levels. To extract the color and texture features from the obtained image channels, a program was coded in MATLAB Software. The coded program called the images and automatically calculated the features after the preprocessing step and saved the features in an Excel file.

Ten color features including the mean, standard deviation, coefficient of variation, kurtosis, skewness, covariance (Eqs. 2–7), maximum, minimum, middle, and mode^22–24,29,30^ were calculated.

2 $${\upmu } = \frac{1}{{{\text{MN}}}}\mathop \sum \limits_{{{\text{i}} = 1}}^{{\text{M}}} \mathop \sum \limits_{{{\text{j}} = 1}}^{{\text{N}}} {\text{P}}\left( {{\text{i}},{\text{ j}}} \right)$$

3 $$\sigma = \left[ {\frac{1}{MN}\mathop \sum \limits_{i = 1}^{M} \mathop \sum \limits_{j = 1}^{N} \left( {P\left( {i,j} \right) - \mu } \right)} \right]^{{{\raise0.7ex\hbox{$1$} \!\mathord{\left/ {\vphantom {1 2}}\right.\kern-\nulldelimiterspace} \!\lower0.7ex\hbox{$2$}}}}$$

4 $$C_{v} = \frac{\sigma }{\mu }$$

5 $$Sk = \frac{1}{{MN\sigma^{3} }}\mathop \sum \limits_{i = 1}^{M} \mathop \sum \limits_{j = 1}^{N} \left[ {\left( {P\left( {i,j} \right) - \mu } \right)} \right]^{3}$$

6 $$Ku = \frac{1}{{MN\sigma^{4} }}\mathop \sum \limits_{i = 1}^{M} \mathop \sum \limits_{j = 1}^{N} \left[ {\left( {P\left( {i,j} \right) - \mu } \right)} \right]^{4}$$

7 $$Cov\left( {X, \, Y} \right) = E((X - u)(Y - u))$$

where: *u*: The mean, *σ*: The standard deviation; *Cv*: The coefficient of variation; *Sk*: The skewness; *Ku*: The kurtosis; *Cov* is the covariance; *M*: The number of rows of the images; *N*: The number of columns of the images; *P (i, j)*: The color values of *i* column and *j* row; *υ = E(Y),*; *µ = E(X).*

Texture features were energy, entropy, contrast, correlation, and homogeneity features (Eqs. 8–12). To extract texture features, firstly the gray-level co-occurrence matrix (GLCM) was obtained for each image channel. The GLCM matrix is a statistical method for examining the texture via considering the spatial relationship between pixels in an image. From the GLCM matrix, five texture features including energy, entropy, contrast, correlation, and homogeneity features^22–24,29^ were calculated.

8 $$Ee = - \mathop \sum \limits_{i = 1}^{M} \mathop \sum \limits_{j = 1}^{N} P_{d}^{2} \left( {i,j} \right)$$

9 $$Et = - \mathop \sum \limits_{i = 1}^{M} \mathop \sum \limits_{j = 1}^{N} P_{d} \left( {i,j} \right)\log P_{d} \left( {i, j} \right)$$

10 $$c = \mathop \sum \limits_{i = 1}^{M} \mathop \sum \limits_{j = 1}^{N} \left( {i - j} \right)^{2} P_{d} \left( {i,j} \right)$$

11 $$Corr = \frac{{\mathop \sum \nolimits_{i = 1}^{M} \mathop \sum \nolimits_{j = 1}^{N} \left( {1 - \mu_{i} } \right)P_{d} \left( {i,j} \right)}}{{\sigma_{i} \sigma_{j} }}$$

12 $$H = \mathop \sum \limits_{i = 1}^{M} \mathop \sum \limits_{j = 1}^{N} \frac{{P_{d} \left( {i,j} \right)}}{{1 + \left| {i - j} \right|}}$$

where: *Ee:* The energy; *H*: The homogeneity; *Et*: The entropy; *C*: The contrast; *Corr*: The correlation; *M*: The number of rows of the images; *N*: The number of columns of the images; *Pd*: The co-occurrence matrix; *Pd (i, j)*: The values at *i* column and *j* row of the co-occurrence matrix; *σi,j*: The standard deviation associated with *i* column and *j* row.

As 19 image channels were obtained and 15 features were extracted from each image channel, totally, 285 features were obtained for each image. These features were analyzed in the next step.

### Analysis

After extracting the image features and saving them in an Excel file, the features were used to develop predicting models. To predict the dust concentration, the extracted features were considered as independent variables, and the dependent variable was the actual dust concentration that was measured by the dust meter.

As the extracted features from the images are compared to select the efficient data to be used for prediction goals^22–24^, the 285 extracted features were compared with each other in the present research. For that, single variable models were developed for each extracted feature based on linear single-variable regression method. Totally, 285 single-variable models were obtained. Also, the predictive accuracy (R^2^) of each model was calculated as a criterion for comparing the features with each other. Those models with higher predictive accuracy have been selected (Fig. 1 and Table 1) as the best single-variable models and then the feature of the selected models was used to develop multivariable models.

A program was coded in MATLAB 2013A software^31,32^ to develop multivariable models. Different multivariable models from 2-variable to 9-variable were developed (Table 2) to find the best predictive model for dust concentration. In the models, the independent variables were the selected features and the dependent variable was actual dust concentration.

## Data Availability

The datasets used during the current study are available from the corresponding author on reasonable request.
